# Tetrabromidocuprates(II)—Synthesis, Structure and EPR

**DOI:** 10.3390/ijms17040596

**Published:** 2016-04-20

**Authors:** André Zabel, Alette Winter, Alexandra Kelling, Uwe Schilde, Peter Strauch

**Affiliations:** 1 Institute of Chemistry, University of Potsdam, Karl-Liebknechtstr.-Str. 24-25, Potsdam D-14476, Germany; azabel@uni-potsdam.de (A.Z.); kelling@uni-potsdam.de (A.K.); uschilde@uni-potsdam.de (U.S.); 2 Engineering Education Research Group, Hamburg University of Technology (TUHH), Am Schwarzberg-Campus 3 (E), Hamburg D-21073, Germany; alette.winter@tuhh.de

**Keywords:** tetrabromidocuprate(II), X-ray structure, electron paramagnetic resonance, copper(II)

## Abstract

Metal-containing ionic liquids (ILs) are of interest for a variety of technical applications, e.g., particle synthesis and materials with magnetic or thermochromic properties. In this paper we report the synthesis of, and two structures for, some new tetrabromidocuprates(II) with several “onium” cations in comparison to the results of electron paramagnetic resonance (EPR) spectroscopic analyses. The sterically demanding cations were used to separate the paramagnetic Cu(II) ions for EPR measurements. The EPR hyperfine structure in the spectra of these new compounds is not resolved, due to the line broadening resulting from magnetic exchange between the still-incomplete separated paramagnetic Cu(II) centres. For the majority of compounds, the principal *g* values (*g*_‖_ and *g*_⊥_) of the tensors could be determined and information on the structural changes in the [CuBr_4_]^2−^ anions can be obtained. The complexes have high potential, e.g., as ionic liquids, as precursors for the synthesis of copper bromide particles, as catalytically active or paramagnetic ionic liquids.

## 1. Introduction

**Scheme 1:**
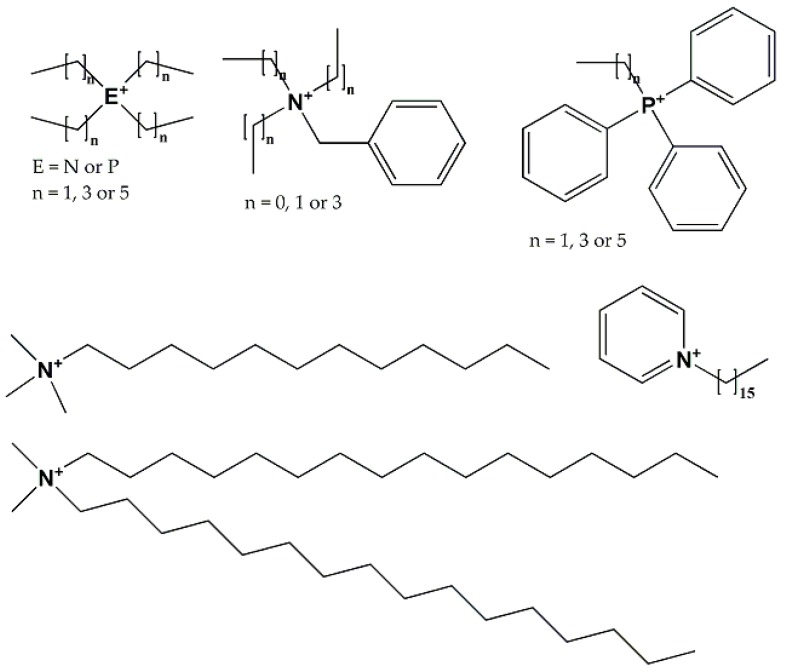
Cations of the [CuBr_4_]^2−^ salts.

Metal-containing ionic liquids (ILs) or ionic liquid crystals (ILCs) are of interest for a variety of technical applications, e.g., as precursors for the synthesis of (nano)particles, magnetic or thermochromic materials [1,2,3] or as extraction agents [4] and catalysts [5,6,7,8]. Tetrahalidometalate complexes exhibit many of these interesting properties. Also liquid-crystalline ILs based on tetrahalidometalates have been reported [9,10,11,12,13]. Some ILs and ILCs combining tetrahalidocuprate dianions with alkylpyridinium cations can serve as precursors for inorganic materials [2,14,15,16,17]. A variety of these metal-containing ionic liquids show interesting electrochemical properties as electrochromy and/or magnetic behaviour [18,19]. The properties of ionic liquid materials such as tuneable acidity, polarity, amphiphilic character, coordinating ability, and miscibility with many compounds might be combined with the unique properties introduced by the metal ions. ILs of this type are also of interest as catalysts, as alternative solvents and even morphology templates for inorganic materials simultaneously. Reports on tetrahalidometalate-based ILs/ILCs and their applications as well as their structural characterization are still relatively rare [2,10,11,12,13,20,21,22,23,24,25,26,27,28,29,30,31,32]. We present the syntheses, electron paramagnetic resonance (EPR) spectra and two X-ray structures of some new tetrabromidocuprate(II) compounds. The following cations with different steric demand were used to separate the paramagnetic Cu(II) ions for the EPR measurements (see Scheme 1): the homogenously substituted tetraalkyl onium cations with varying chain lengths tetraethylammonium, (Et_4_N^+^), tetrabutylphosphonium, (Bu_4_P^+^), tetrahexylammonium, (Hex_4_N^+^), the benzyl/alkyl substituted ammonium cations benzyltrimethylammonium, (BzlMe_3_N^+^), benzyltriethylammonium, (BzlEt_3_N^+^), benzyltributylammonium, (BzlBu_3_N^+^), the alkyltriphenylphosphonium cations ethyltriphenylphosphonium, (EtPh_3_P^+^), hexyltriphenylphosphonium, (HexPh_3_P^+^) and the long chain cations dodecyltrimethylammonium, (C_12_H_25_Me_3_N^+^), hexadecylpyridinium, (C_16_H_33_-py^+^), hexadecyldimethylammonium, (C_16_H_33_)_2_Me_2_N^+^), giving raise to lamellar structures [33]. It is known that tetrahalidocuprate(II)-ions present a high structural flexibility. Thus, the complex anion adopts coordination geometries between square-planar and tetrahedral. Recently we have shown that there is a correlation of EPR parameters and the isotropic or averaged *g*-values with the degree of geometrical distortion in the coordination sphere of tetrahalidocuprates expressed by the *cis*-angle, the average of the four smallest X-Cu-X angles of the tetrahalidocuprate dianion [33,34,35].

## 2. Results and Discussion

### 2.1. X-ray Crystallography

Table 1 summarizes the crystallographic data and refinement parameters for the structures of (BzlEt_3_N)_2_[CuBr_4_] (**5**) and (HexPh_3_P)_2_[CuBr_4_] (**8**).

### 2.2. Bis(benzyltriethylammonium)tetrabromidocuprate(II) (**5**)

**Figure 1:**
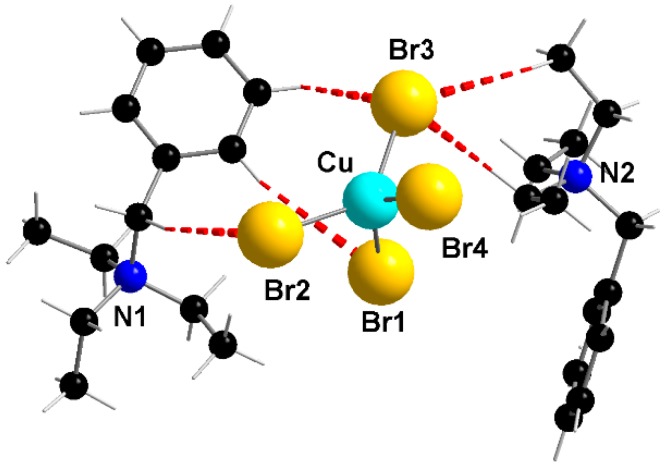
Molecular structure of (BzlEt_3_N)_2_[CuBr_4_] (**5**), hydrogen contacts are presented as fragmented lines.

(BzlEt_3_N)_2_[CuBr_4_] (**5**) crystallizes in the monoclinic space group *P*2_1_/*c* with four formula units per unit cell. The corresponding lattice parameters are *a* = 15.1019(5) Å, *b* = 11.6664(5) Å, *c* = 17.5517(6) Å, and *β* = 99,166(3)°. The complex dianion has a distorted tetrahedral geometry with Br–Cu–Br angles between 98.2° and 131.8° (see Table 2), the resulting *cis*-angle, the average of the four smallest angles, is 99.8°. The shortest Cu···Cu distances are 9.12 and 9.44 Å. Figure 1 shows the molecular structure of (BzlEt_3_N)_2_[CuBr_4_] (**5**). The structure is stabilized by a series of hydrogen bonds (see Table 3).

### 2.3. Bis(hexyltriphenylphosphonium)tetrabromidocuprate(II) (**8**)

**Figure 2:**
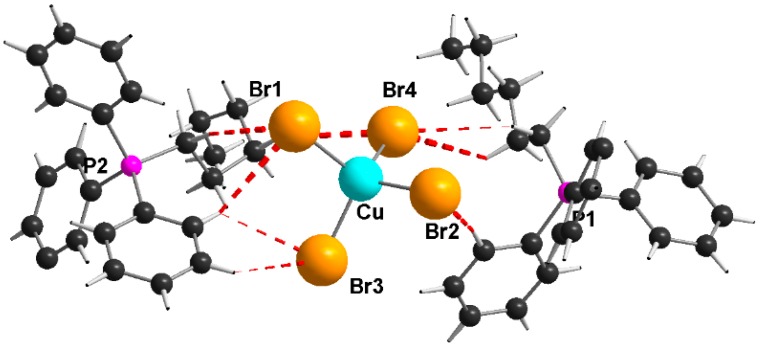
Molecular structure of (HexPh_3_P)_2_[CuBr_4_] (**8**), hydrogen contacts are presented as fragmented lines.

(HexPh_3_P)_2_[CuBr_4_] (**8**) crystallizes in the monoclinic space group *P*2_1_/*n* with *Z* = 4. The corresponding lattice parameters are *a* = 10.7410(3) Å, *b* = 22.4775(9) Å, *c* = 19.6455(6) Å, and *β* = 99,807(2)°. This complex dianion has also a distorted tetrahedral geometry with Br–Cu–Br angles between 100.4° and 124.8° (Table 4), with a resulting *cis*-angle of 103.3°. The shortest Cu···Cu distances are 10.62 and 10.74 Å. Figure 2 shows the molecular structure of (HexPh_3_P)_2_[CuBr_4_] (**8**). The structure is stabilized by a variety of hydrogen contacts (Table 5).

### 2.4. Electron Paramagnetic Resonance (EPR) Spectroscopy

**Figure 3:**
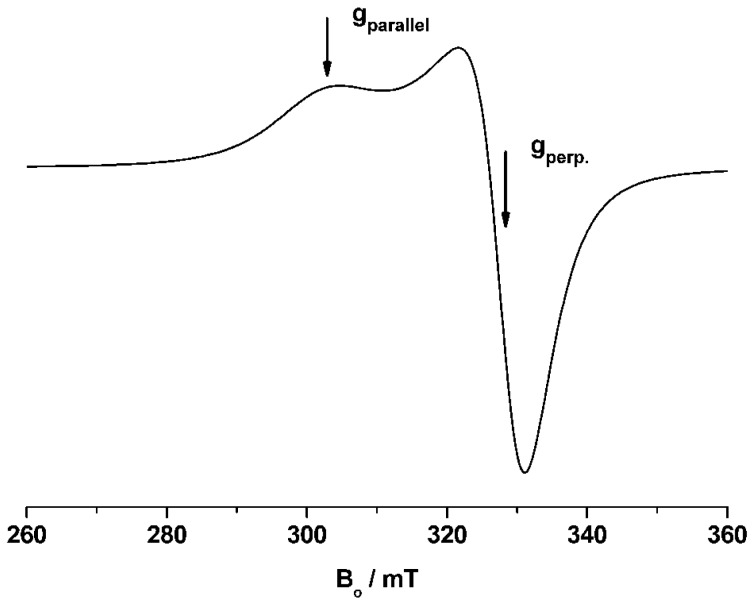
Electron paramagnetic resonance (EPR) spectrum of (EtPh_3_P)_2_[CuBr_4_] (**7**) at 150 K.

Figure 3 shows a spectrum of (EtPh_3_P)_2_[CuBr_4_] (**7**) at 150 K, characteristic for most of the recorded spectra. In general the spectra are of poor resolution, due to the magnetic interactions between the paramagnetic centers and no hyperfine structure can be observed. Some of the spectra are of axial symmetry and *g*_‖_ as well as *g*_⊥_ can be determined; for a few samples only an isotropic signal (*g*_iso_) could be extracted. The EPR data are shown in Table 6. The *g*_av_-values correspond to the isotropic *g*_iso_-value of liquid systems as long as strong solvents are excluded (e.g., solutions or ionic liquids).

The averaged *g*-values *g*_av_ for axial symmetry are calculated by the following expression:

(1) $$g_{av}=\frac{g_{II}+2\times g_{⊥}}{3}$$

For rhombic symmetry the averaged *g*-values *g*_av_ are calculated as follows:

(2) $$g_{av}=\frac{g_{1}\;+\;g_{2}+g_{3}}{3}$$

**Figure 4:**
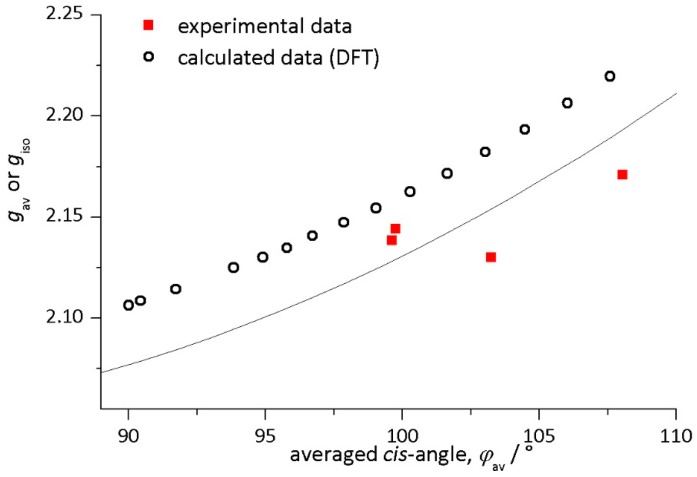
Correlation of EPR parameter (*g*_av_/*g*_iso_) with the coordination geometry (averaged *cis*-angle) including the four new data sets from Table 6 (squares); the circles represent the calculated data (DFT).

The variation of the structural parameter (***ϕ***_av_) reflects the degree of structural flexibility of the tetrahalidocuprate(II) moiety. With a series of known X-ray structures combined with EPR parameters of tetrabromidocuprates(II) it could be possible to classify the degree of distortion between square planar and tetrahedral geometries, as well as those of complexes with unknown structures as was recently shown for tetrabromidocuprates(II) [34]. With larger the *cis*-angles the *g*_av_ or the isotropic *g*_iso_-values have a tendency to increase (see Figure 4). This is also supported by calculated values from DFT calculations.

**Figure 5:**
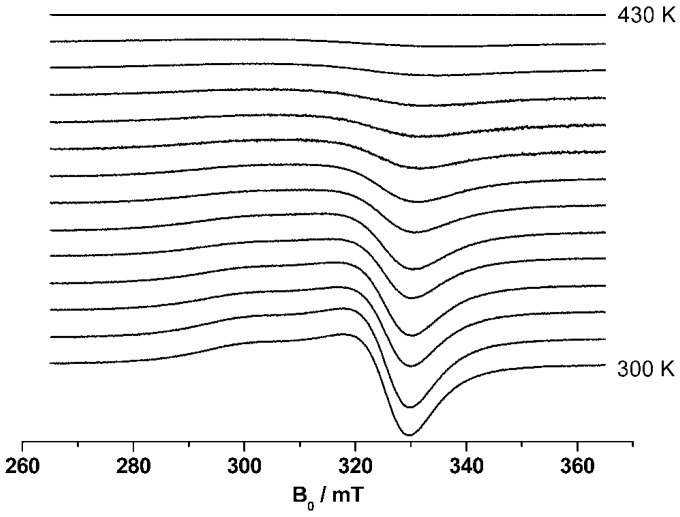
Temperature dependent EPR spectra of (EtPh_3_P)_2_[CuBr_4_] (**7**) recorded in the temperature range from 300 to 430 K (10 K steps).

**Figure 6:**
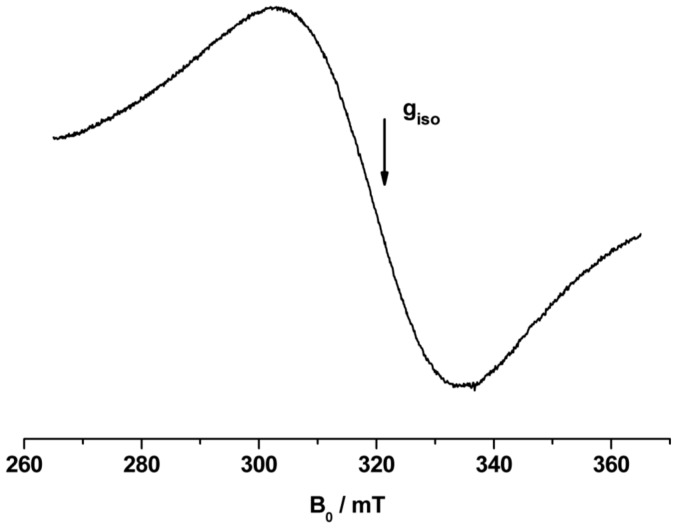
EPR spectrum of (EtPh_3_P)_2_[CuBr_4_] (**7**) at 150 K directly after the thermal treatment; only an isotropic signal remains.

**Figure 7:**
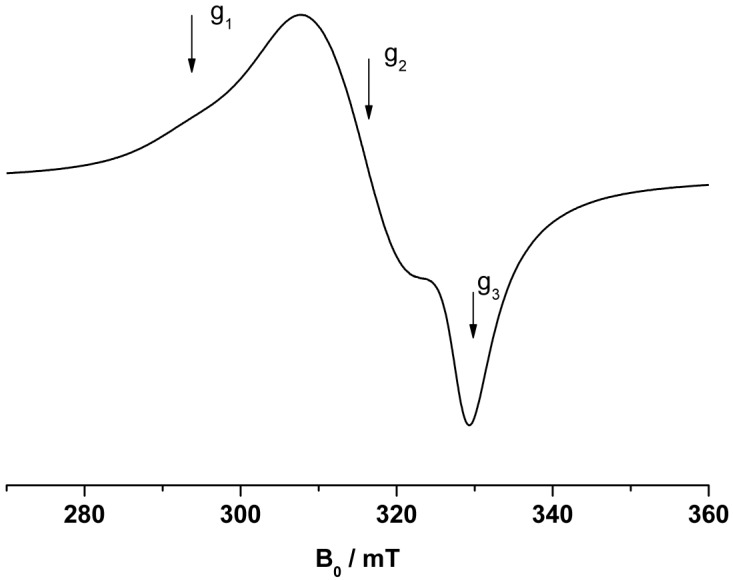
EPR spectrum of (C_12_H_25_Me_3_N)_2_[CuBr_4_] (**9**) at 110 K.

Figure 5 shows the temperature-dependent EPR spectra of (EtPh_3_P)_2_[CuBr_4_] (**7**) recorded in a temperature range from 300 to 430 K. The signal intensity decreases with rising temperature until it is fully distinct, due to spin saturation effects. This thermal cycle is completely reversible without any signs of decomposition. Interestingly, after cooling to room temperature, only an isotropic EPR signal remains (Figure 6), which returned to the starting axial symmetric spectrum after a couple of days (Figure 7). Obviously the re-crystallization process is kinetically enhanced. Compounds of this type might be useful as ionic liquids for higher temperatures.

The EPR spectrum of (C_12_H_25_Me_3_N)_2_[CuBr_4_] (**9**) at 110 K (Figure 7) reflects a rhombic symmetry of the *g*-tensor with three different *g*-values and indicates a change in coordination geometry or a possible lamellar structure.

### 2.5. Differential Scanning Calorimetry (DSC)

**Figure 8:**
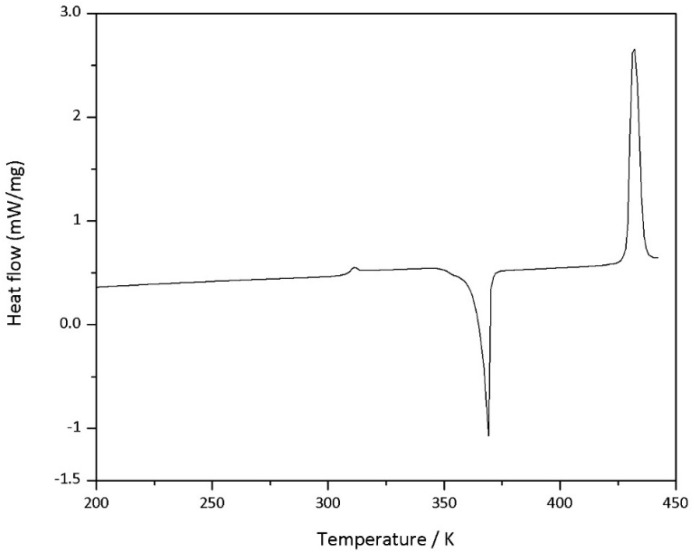
Second DSC scan from (EtPh_3_P)_2_[CuBr_4_] (**7**).

(EtPh_3_P)_2_[CuBr_4_] (**7**) shows a glass transitions at 306 K, a melting point at 432 K and a cold crystallization by 369 K. Figure 8 shows the second heat run of the compound. The third measurement comes to the same result as the second. It can be concluded that the compound has a temperature reversibility at least up to 443 K. The results of the differential scanning calorimetry of (**7**) are in good agreement with the data from EPR-spectroscopy.

## 3. Materials and Methods

### 3.1. Methods

The melting points were determined using a Mikroheiztisch Boetius (VEB Wägetechnik, Radebeul, DDR). Elemental analyses were carried out on an Elementar Vario EL III analyzer (elementar Analysensysteme GmbH, Hanau, Germany). Infrared spectra were recorded on a Perkin-Elmer type 16PC FT-IR spectrophotometer (Perkin-Elmer GmbH, Überlingen, GErmany) between 4000 and 400 cm^−1^ as KBr-pellets (reference KBr). The measurements of the magnetic susceptibility were performed with a Magnetic Susceptibility Balance-Auto from Johnson Matthey GmbH (Matthey GmbH, Cambridge, UK) at room temperature, for diamagnetic correction the increment system of Pascal and Pacault [38] was applied. EPR spectra were recorded at 9.4 GHz (X-band) using a Bruker CW Elexsys E 500 spectrometer (Bruker BioSpin GmbH, Rheinstetten, Germany)

For X-ray structure determinations, the crystals were embedded in perfluoropolyalkylether oil and mounted on a glass fibre (**5**) or within a MicroGripper (**8**). For structure analysis of (**5**), the intensity data were collected at 210 K using an Imaging Plate Diffraction System IPDS-2 (Stoe, Darmstadt, Germany) with graphite monochromatized Mo-*K*α radiation (λ = 0.71073 Å) at 50 kV and 40 mA. The data collection for (**8**) was performed on a StadiVari diffractometer (Stoe, Darmstadt, Germany) equipped with a four-circle goniometer (open Eulerian cradle), a Genix Microfocus X-ray source (Mo) with a graded multilayer mirror and a Pilatus 200 K detector (Dectris, Baden-Daettwil, Switzerland). The data were corrected for absorption as well as for Lorentz polarization and extinction effects using the program X-Area (Stoe, 2004) [39]. The structures were solved by direct methods using SHELXS-2013/1 [40] and refined by full-matrix least squares on *F*^2^ using the program SHELXL-2014/7 [41]. All non-hydrogen atoms were refined anisotropically. The hydrogen atoms were calculated in their expected positions and refined with a riding model. CCDC 1459578 (**5**) and CCDC 1459612 (**8**) contain the supplementary crystallographic data for this paper. These data are provided free of charge by The Cambridge Crystallographic Centre (Cambridge, UK).

Differential scanning calorimetry (DSC) measurements were performed with a DSC 214 Polyma (Netzsch GmbH & Co. KG, Selb, Germany) by NETZSCH operating with a scan rate of 5–10 °C·min^−1^ under a nitrogen flow.

### 3.2. Chemicals

The following chemicals were used without further purification. Copper(II) bromide (99%), ethanol anhydrous (99%), n-hexane (96%), potassium bromide (Uvasol, Merk KGaA, Darmstadt, Germany, for IR spectroscopy) tetraethylammonium bromide (98%), tetrabutylphosphonium bromide (99%), tetrahexylammonium bromide (99%), benzyltrimethylammonium bromide (98%), benzyltriethylammonium bromide (98%), benzyltributylammonium bromide (99%), ethyltriphenylphosphonium bromide (98%), hexyltriphenylphosphonium bromide (>99%), dodecyltrimethylammonium bromide (>98%), hexadecylpyridinium bromide (98%), and dihexadecyldimethylammonium bromide (99%).

### 3.3. Syntheses

#### 3.3.1. General Preparation

In general, tetrabromidocuprate(II) complexes can be achieved by different procedures [42,43,44]. In the current work, the [CuBr_4_]^2−^ moiety was synthesized according to a protocol by N. S. Gill and R. S. Nyholm [44]: an ethanolic solution of a stoichiometric amount of CuBr_2_ was added to the respective bromide salt of the cation dissolved in a minimum volume of ethanol. The reaction mixture was stirred for one hour at room temperature. The product was precipitated by evaporating the solvent.

#### 3.3.2. Bis(tetraethylammonium)tetrabromidocuprate(II), (Et_4_N)_2_[CuBr_4_] (**1**)

Compound (**1**) was synthesized according to an already published protocol [36].

A solution of 1.5 mmol (0.32 g) of tetraethylammonium bromide in 3 mL of ethanol was mixed with a solution of 0.5 mmol (0.11 g) of copper(II) bromide in 10 mL ethanol. The solution was stirred one hour at room temperature. The solvent was removed and a violet powder was received, filtered off and dried.

Melting point: 241–242 °C. Yield: 0.22 g (67%). Elemental analysis calculated for C_16_H_40_N_2_CuBr_4_ (643.66): C 29.85, H 6.26, N 4.35 (%); found: C 29.80, H 6.20, N 4.34 (%). IR (KBr, cm^−1^): 3443 s, 2976 m, 2921 m, 2852 wm, 1628 wm, 1478 s, 1402 m, 1307 w, 1183 m, 1032 m, 1006 m, 793 m.

(IR: s = strong, ms = medium strong, m = medium, wm = weak medium, w = weak).

#### 3.3.3. Bis(tetrabutylphosphonium)tetrabromidocuprate(II), (Bu_4_P)_2_[CuBr_4_] (**2**)

Solutions of 0.5 mmol (0.11 g) copper(II) bromide and 1.0 mmol (0.32 g) tetrabutylphosphonium bromide, each dissolved in 2 mL ethanol, were combined and stirred at room temperature for 1 h. The resulting violet precipitate is filtered off and dried.

Melting point: 49–52 °C. Yield: 0.38 g (84%). Elemental analysis calculated for C_32_H_72_P_2_CuBr_4_ (902.13): C 42.60, H 8.04 (%), found: C 42.08, H 8.16 (%). IR (KBr, cm^−1^): 2928 s, 2891 ms, 2870 ms, 1463 ms, 1407 m, 1375 m, 1237 w, 1098 m, 968 w, 920 m, 722 m. *μ*_eff_ = 1.5 B.M.

#### 3.3.4. Bis(tetrahexylammonium)tetrabromidocuprate(II), (Hex_4_N)_2_[CuBr_4_] (**3**)

A solution of 1.0 mmol (0.22 g) CuBr_2_ in 5 mL ethanol was added to a solution of 2.0 mmol (0.87 g) tetrahexylammonium bromide in 5 mL ethanol. The mixed solution was stirred at room temperature for 1 h. The resulting violet precipitate was filtered off and dried.

Melting point: 93–95 °C. Yield: 0.58 g (53%). Elemental analysis calculated for C_48_H_104_N_2_CuBr_4_ (1092.51): C 52.77, H 9.60, N 2.56 (%), found: C 53.06, H 9.70, N 2.67 (%). IR (KBr, cm^−1^): 2928 s, 2553 m, 1634 w, 1480 m, 1383 mw, 1050 w, 728 w. *μ*_eff_ = 1.5 B.M.

#### 3.3.5. Bis(benzyltrimethylammonium)tetrabromidocuprate(II), (BzlMe_3_N)_2_[CuBr_4_] (**4**)

Compound (**4**) was also synthesized according to an already published procedure [37].

To a solution of 0.5 mmol (0.11 g) of CuBr_2_ in 3.5 mL methanol a solution of 1.0 mmol (0.23 g) benzyltrimethylammonium bromide, dissolved in 1.5 mL methanol, was added. The mixture was stirred for one hour at room temperature. After a short while the complex precipitated as purple crystals.

Melting point: 173–175 °C. Yield: 0.35 g (72%). Elemental analysis calculated for C_20_H_32_N_2_CuBr_4_ (969.51): C 35.16, H 4.72, N 4.10 (%); found: C 35.09, H 4.71, N 4.17 (%). IR (KBr, cm^−1^): 3017 m, 1585 w, 1485 s, 1476 s, 1411 m, 1218 m, 989 m, 974 m, 887 s, 780 s, 703 s. *μ*_eff_ = 1.4 B.M.

#### 3.3.6. Bis(benzyltriethylammonium)tetrabromidocuprate(II), (BzlEt_3_N)_2_[CuBr_4_] (**5**)

0.5 mmol (0.11 g) of CuBr_2_ dissolved in 3 mL HBr was heated under reflux for 0.5 h. To this copper solution a solution of 1.0 mmol (0.27 g) BzlEt_3_NBr and 2 mL methanol was slowly added. The mixture was stirred for one hour at room temperature. The solvent was reduced and purple crystals were obtained by covering the remaining solution with *N*-hexane.

Melting point: 110–112 °C. Yield: 0.21 g (54%). Elemental analysis calculated for C_26_H_44_N_2_CuBr_4_ (767.7): C 40.67, H 5.78, N 3.65 (%); found: C 40.56, H 5.76, N 3.69 (%). IR (KBr, cm^−1^): 2983 m, 1583 w, 1450 s, 1402 ms, 1372 w, 1171 w, 1154 m, 1027 m, 1005 m, 787 m,756 s, 705 s. *μ*_eff_ = 1.6 B.M.

#### 3.3.7. Bis(benzyltributylammonium)tetrabromidocuprate(II), (BzlBu_3_N)_2_[CuBr_4_] (**6**)

A solution of 1.0 mmol (0.22 g) CuBr_2_ in 5 mL ethanol was added to a solution of 2.0 mmol (0.71 g) benzyltributylammonium bromide in 5 mL ethanol. The mixed solution was stirred at room temperature for 1 h. The resulting violet precipitate was filtered off and dried.

Melting point: 58–60 °C. Yield: 0.59 g (63%). Elemental analysis calculated for C_38_H_68_N_2_CuBr_4_ (936.12): C 48.75, H 7.32, N 2.99 (%), found: C 48.90, H 7.48, N 3.12 (%). IR (KBr, cm^−1^): 2959 s, 2871 m, 1654 w, 1561 w, 1474 m, 1458 m, 1380 mw, 1212 w, 869 mw, 725 m, 704 m. *μ*_eff_ = 1.5 B.M.

#### 3.3.8. Bis(ethyltriphenylphosphonium)tetrabromidocuprate(II), (EtPh_3_P)_2_[CuBr_4_] (**7**)

The synthesis of (EtPh_3_P)_2_[CuBr_4_] as follows: 0.5 mmol (0.11 g) copper(II) bromide and 1.0 mmol (0.37 g) EtPh_3_PBr solved even in 2 mL ethanol. The combined solutions are stirred for one hour at room temperature. A violet powder was obtained.

Melting point: 135–137 °C. Yield: 0.23 g (48%). Elemental analysis calculated for C_40_H_40_P_2_CuBr_4_ (964.90): C 49.74, H 4.17 (%), found: C 49.74, H 4.19 (%). IR (KBr, cm^−1^): 2922 m, 1586 m, 1483 m, 1438 s, 1113 s, 996 m, 780 w, 739 s, 691 s, 531 s, 510 s, 482 m. *μ*_eff_ = 1.6 B.M.

#### 3.3.9. Bis(hexyltriphenylphosphonium)tetrabromidocuprate(II), (HexPh_3_P)_2_[CuBr_4_] (**8**)

A solution of 1.0 mmol (0.22 g) copper(II) bromide in 5 mL ethanol is added to 2.0 mmol (0.85 g) hexyltriphenylphosphonium bromide dissolved in 5 mL ethanol. The solution was stirred for 1 h at room temperature. The formed violet precipitate was filtered off and dried. Purple crystals were obtained by covering the remaining solution with n-hexane for slowly interdiffusion.

Melting point: 102–103 °C. Yield: 0.65 g (60%). Elemental analysis calculated for C_48_H_56_P_2_CuBr_4_ (1078.04): C 53.47, H 5.24 (%), found: C 53.05, H 4.97 (%). IR (KBr, cm^−1^): 2956 m, 2923 m, 2758 m, 1585 w, 1485 w, 1436 s, 1113 s, 724 s, 689 s, 533s, 498 ms. *μ*_eff_ = 1.6 B.M.

#### 3.3.10. Bis(dodecyltrimethylammonium)tetrabromidocuprate(II), (C_12_H_25_Me_3_N)_2_[CuBr_4_] (**9**)

A solution of 1.0 mmol (0.22 g) of CuBr_2_ in 5 mL ethanol and a solution of 2.0 mmol (0.62 g) C_12_H_25_Me_3_NBr in 5 mL ethanol were combined and stirred at room temperature for 1 h. The resulting violet precipitate was filtered off and dried.

Melting point: 94–95 °C. Yield: 0.56 g (85%). Elemental analysis calculated for C_30_H_48_N_2_CuBr_4_ (662.23): C 42.89, H 8.16, N 3.34 (%), found: C 43.19, H 8.23, N 3.37 (%). IR (KBr, cm^−1^): 2922 ms, 2851 m, 1632 w, 1469 s, 966 ms, 909 m, 722 m. *μ*_eff_ = 1.5 B.M.

#### 3.3.11. Bis(hexadecylpyridinium)tetrabromidocuprate(II), (C_16_-py)_2_[CuBr_4_] (**10**)

A solution of 0.5 mmol (0.11 g) copper(II) bromide in 10 mL ethanol is added to 1.0 mmol (0.40 g) hexadecylpyridinium bromide dissolved in 3 mL ethanol. The solution was stirred for 1 h at room temperature. The obtained violet precipitate was filtered off and dried.

Melting point: 73–74 °C. Yield: 0.15 g (30%). Elemental analysis calculated for C_42_H_76_N_2_CuBr_4_ (992.23): C 50.84, H 7.72, N 2.82 (%), found: C 47.67, H 7.20, N 2.95 (%). IR (KBr, cm^−1^): 3446 wm, 3053 w, 2918 s, 2850 m, 1633 wm, 1485 m, 1468 wm, 1175 w, 768 w, 721 w, 679 w. *μ*_eff_ = 1.6 B.M.

#### 3.3.12. Bis(dihexadecyldimethylammonium)tetrabromidocuprate(II), (C_16_H_33_)_2_Me_2_N)_2_[CuBr_4_] (**11**)

A solution of 1.0 mmol (0.58 g) dihexadecyldimethylammonium bromide in 3 mL ethanol was slowly added to a solution of 0.5 mmol (0.11 g) copper(II) bromide in 3 mL ethanol. The mixture was stirred at room temperature for 1 h. The formed violet precipitate was filtered off and dried.

Melting point: 36–38 °C. Yield: 0.67 g (98%). Elemental analysis calculated for C_68_H_144_N_2_CuBr_4_ (1373.05): C 59.48, H 10.57, N 2.04 (%), found: C 59.11, H 10.89, N 2.15 (%). IR (KBr, cm^−1^): 2919 m, 2851 m, 1628 w, 1469 s, 1376 w, 1054 w, 990 w, 968 w, 878 w, 718 m.

## 4. Conclusions

Some of the compounds in this series are real ionic liquids—(**2**), (**3**), (**6**), (**10**) and (**11**)—with melting points below 100 °C, or very close to it—(**5**) and (**8**). All the reported compounds are thermally stable up to at least 430 K. This thermal cycle is completely reversible without any signs of decomposition. Interestingly, after cooling to room temperature, for (**7**) only an isotropic EPR signal remains, which returned to the axial symmetric spectrum after a couple of days. That means the re-crystallizing process is kinetically enhanced. Compounds of this type might be useful as ionic liquids for higher temperatures.

Two compounds, (**5**) and (**8**), could be structurally characterized by X-ray structure analysis. The structures are stabilized by a variety of hydrogen contacts between the [CuBr_4_]^2−^ anions and corresponding onium cations, responsible for the coordination geometry of the tetrahalidocuprates. The EPR parameters also reflect the degree of structural flexibility of the tetrahalidocuprate(II) moiety. With a series available data sets of known X-ray structures combined with EPR parameters of tetrabromidocuprates(II) it is possible to classify the degree of the distortion coordination sphere between square planar and tetrahedral geometries, as was recently shown for tetrabromidocuprates(II) [34].

## Figures and Tables

**Table 1:** Crystallographic data and refinement parameters for (**5**) and (**8**).

Compound	(BzlEt_3_N)_2_[CuBr_4_] (5)	(HexPh_3_P)_2_[CuBr_4_] (8)
Empirical formula	C_26_H_44_N_2_CuBr_4_	C_48_H_56_P_2_CuBr_4_
*M*_W_/g·mol^−1^	767.81	1078.04
Crystal description	purple plate	purple needle
Crystal size/mm	0.7 × 0.5 × 0.1	0.8 × 0.2 × 0.1
Crystal system	monoclinic	monoclinic
Space group	*P*2_1_*/c*	*P*2_1_*/n*
*a*/Å	15.1019(5)	10.7410(3)
*b*/Å	11.6664(5)	22.4775(9)
*c*/Å	17.5517(6)	19.6455(6)
*α*/°	90	90
*β*/°	99.166(3)	99.807(2)
*γ*/°	90	90
*V*/Å^3^	3052.9(2)	4673.7(3)
*Z*	4	4
*F*(000)	1532	2172
Density/mg·m^−3^	1.67	1.532
*μ*/mm^−1^	5.97	3.987
Θ range/°	2.10–25.00	2.15–24.998
*R*_int_	0.1338	0.0740
Reflections measured	38,767	73,469
Reflections independent	5372	8223
Parameters	299	496
*R*_1_*/wR*_2_ (*I* > 2σ(*I*))	0.0370/0.0893	0.0480/0.1183
*R*_1_*/wR*_2_ (all data)	0.0529/0.0975	0.0711/0.1293
Goodness of fit	1.015	1.031
Max. difference peak/hole/e Å^−3^	0.71/−0.83	2.231/−1.220

**Table 2:** Selected bond lengths and bond angles of (BzlEt_3_N)_2_[CuBr_4_] (**5**).

Bond Lengths/Å		Bond Angles/°	
Cu1–Br1	2.3981(7)	Br1–Cu1–Br2	99.73(2)
		Br1–Cu1–Br3	98.38(2)
		Br1–Cu1–Br4	130.80(3)
Cu1–Br2	2.3747(7)	Br2–Cu1–Br3	131.84(3)
Cu1–Br3	2.3918(7)	Br2–Cu1–Br4	98.19(2)
Cu1–Br4	2.3984(7)	Br3–Cu1–Br4	102.80(2)

**Table 3:** Hydrogen contacts for (BzlEt_3_N)_2_[CuBr_4_] (**5**).

C–H∙∙∙Br	H∙∙∙Br/Å	C∙∙∙Br/Å	Angles C–H∙∙∙Br/°
C1–H1A···Br2 ^i^	2.93	3.568(4)	124.2
C2–H2A···Br2	3.09	3.955(6)	151.1
C6–H6A···Br2 ^ii^	2.82	3.742(6)	161.1
C6–H6A···Br1 ^ii^	3.01	3.621(6)	132.2
C7–H7A···Br2 ^ii^	2.99	3.935(4)	164.6
C7–H7B···Br3	3.04	3.982(5)	165.4
C14–H14*A*···Br1 ^iii^	3.08	3.564(4)	112.3
C14–H14B···Br3	2.92	3.720(5)	140.3
C16–H16A···Br4	3.02	3.849(5)	144.6
C16–H16B···Br1	2.96	3.868(5)	156.9
C20–H20A···Br1 ^ii^	3.08	3.982(4)	154.6

Symmetry codes: (i) 1 − x, 2 − y, 1 − z; (ii) 1 − x, 0.5 + y, 0.5 − z; (iii) −x, −0.5 + y, 0.5 − z.

**Table 4:** Selected bond lengths and bond angles for (HexPh_3_P)_2_[CuBr_4_] (**8**).

Bond Lengths/Å		Bond Angles/°	
Cu1–Br1	2.4500(9)	Br1–Cu1–Br2	120.66(4)
		Br1–Cu1–Br3	103.25(3)
		Br1–Cu1–Br4	104.45(3)
Cu1–Br2	2.3867(9)	Br2–Cu1–Br3	104.95(3)
Cu1–Br3	2.4207(9)	Br2–Cu1–Br4	100.39(3)
Cu1–Br4	2.3677(9)	Br3–Cu1–Br4	124.76(4)

**Table 5:** Hydrogen bond geometry for (HexPh_3_P)_2_[CuBr_4_] (**8**).

C–H∙∙∙Br	H∙∙∙Br/Å	C∙∙∙Br/Å	Angles C–H∙∙∙Br/°
C12–H12∙∙∙Br2	2.82	3.744(6)	166.4
C19–H19B∙∙∙Br3 ^iii^	3.10	4.064(5)	166.4
C26–H26∙∙∙Br1	3.13	3.840(5)	133.8
C26–H26∙∙∙Br3	3.07	3.718(6)	127.2
C32–H32∙∙∙Br4 ^ii^	2.90	3.673(5)	140.8
C36–H36∙∙∙Br2 ^i^	2.84	3.776(5)	176.7
C43–H43B∙∙∙Br1	2.88	3.834(5)	165.9
C43–H43A∙∙∙Br2 ^i^	3.06	4.000(6)	161.1

Symmetry codes: (i) 0,5 + x, 0.5 − y, 0.5 + z; (ii) 1 + x, y, z; (iii) 1 − x, y, z.

**Table 6:** Experimental *g*-values (*g*_‖_, *g*_⊥_ and *g*_iso_), calculated values *g*_av_ and known *cis*-angles (***ϕ***_av_) for this series of tetrabromidocuprates(II).

Cation/Compound	*g*_‖_	*g*_⊥_	*g*_iso_	*g*_av_	*ϕ*_av_/°	Reference (*ϕ*_av_)
Et_4_N^+^/(**1**)	-	-	2.18 ^a^	-	108.06 *	[36]
Bu_4_P^+^/(**2**)	-	-	2.089	-	-	
Hex_4_P^+^/(**3**)	2.34 ^a^	2.079	2.11 ^a^	2.166	-	
BzlMe_3_N^+^/(**4**)	2.221	2.058	2.101	2.109	99.64 *	[37]
BzlEt_3_N^+^/(**5**)	-	-	2.11 ^a^	-	99.77 *	
BzlBu_3_P^+^/(**6**)	-	-	2.108	-	-	
EtPh_3_P^+^/(**7**)	2.217	2.062	-	2.114	-	
BuPh_3_P^+^/(**8**)	2.280	2.069	-	2.139	-	
HexPh_3_P^+^/(**9**)	-	-	2.13 ^a^	-	103.26 *	
(C_12_H_25_)Me_3_N^+^/(**10**)	2.26 ^a^, 2.124, 2.040			2.141	-	
(C_16_H_33_)py^+^/(**11**)	-	-	2.15 ^a^	-	-	
(C_16_H_33_)_2_Me_2_N^+^/(**12**)	2.28 ^a^	2.079	-	2.146	-	

Experimental errors: *g*-values: ±0.005; ^a^ *g*: ±0.01; *: new added EPR/structure data sets.

## Abbreviations

DFT	Discrete Fourier Transform
EPR	Electron Paramagnetic Resonance
DSC	Differential Scanning Calorimetry
